# Book Review: The Imagination of Experiences. Musical Invention, Collaboration, and the Making of Meanings

**DOI:** 10.3389/fpsyg.2021.747876

**Published:** 2021-10-05

**Authors:** Stephan Thomas Vitas

**Affiliations:** University of Maryland, College Park, College Park, MD, United States

**Keywords:** music, composition, interpretation, cognition, perception

In this highly condensed volume, Alan Taylor, both scholar and performer in music, envisions three principal activities to this particular discipline—creation (composer), performance (musician), interpretation (listener), each a different function of imagination, emphasizing the perception of the new, a skill not limited to exceptional people and stressing the need for one's engagement within the world rather than withdrawal into workshop solitude. He draws a perspective with the thread he weaves through a compendium of research, extrapolation of conclusions from the literature and his own experience of music as performer, conductor, and composer.

He examines current trends to present creativity as a skill available to more of the general population than heretofore supposed. Affirming his disbelief in the archetype of lone genius composer, he considers a more democratic origin of multiple sources of inspiration from the environs which the creator inhabits, relying on application of acquired skills over spontaneous genius inspired from within or above. “Their pieces are not timeless masterworks created by geniuses who cannot be questioned, but historically situated art which we can appreciate more fully through a better understanding of its context.” The composer's concept of his piece changes with styles of performance from its debut onward in time. Its origin results from his subconscious elaborating on input from his environment, not necessarily generating its own fresh sounds.

The author stresses the dialogue of influences from the external world of experience that recombines within the subconscious and puts forth its conclusions to be elaborated by well-practiced creative skills. Our interactive repartee with our fellows engenders feelings that reinforce incoming information which is accrued and held in store for memory to add to imagination. Interaction is the key, not passive absorption. The subtle factor is that our “….high level of ability is the result not of superior analytical intelligence but of thinking provoked by a superior ability to sense, experience, and imagine.” For him the learning and imagining processes direct our creation of understandings, which we develop by internal dialogue into creative output. The initial idea generates in the subconscious, emerges into consciousness, where it is considered, evaluated in the subconscious, then elaborated.

Taylor proceeds to consider the barriers inherent in so personal an activity as musical imagination when shared with collaborating creators, detailing different styles of cooperative ventures.

Finally, he details the process of the imaginations of listeners in discerning meanings different for each or approaching those of the composer. Ambiguity is inherent in artistic output, therefore intentionality is neither readily discernible nor accurately definable—perhaps not even necessary. Still, the question of the “meaning” of a piece haunts all discussion of the arts and music no less. Meaning is idiosyncratic to each listener, who infers from emotional and cognitive reactions of “semantic indeterminacy,” even among listeners from similar cultural backgrounds. Music offers no universal code to be broken, but rather a reflection of more general analogies to life experiences. Embodied engagement recalls to cognition previous perceptions, sensations, emotions, real, or imagined.

Dr. Taylor is of the opinion that the perception of responses to music emerge more from the listener's internal history than as a result of specific signals from the score itself. He notes the argument that mirror neurons may be thought to comprise a mechanism conveying perception through experience and into memory, but that this conclusion, as with any concerning hemispheric specialization, require further refinement. He suggests that music's meaning may be an analog of human experience, its perception holistic to the right hemisphere and interpretive by the left—percept and semantics combined, the listeners being “….active agents who construct their own sense of meaning” through the mechanism of their imagination.

Though aimed at a broad audience, this work offers a concise operational overview as foundation material for laboratory and field researcher in music mechanisms and neuroaesthetics from which to design exploratory investigations into variables of generative and interpretive processes, and, equally, anatomical examination of neurological subsystems whose dendritic tracts are known to be near in number to those which mediate language within the cortex. He ties together the totality of his concepts with precise introduction and conclusion sections to each chapter, adding substantial bibliographies to each as well.

In presenting his concise (100 page) analysis of imagination mechanisms in music, Dr. Taylor offers an altogether essential and noteworthy resource to generate further discussion and complex research.

## Author Contributions

The author confirms being the sole contributor of this work and has approved it for publication.

## Conflict of Interest

The author declares that the research was conducted in the absence of any commercial or financial relationships that could be construed as a potential conflict of interest.

## Publisher's Note

All claims expressed in this article are solely those of the authors and do not necessarily represent those of their affiliated organizations, or those of the publisher, the editors and the reviewers. Any product that may be evaluated in this article, or claim that may be made by its manufacturer, is not guaranteed or endorsed by the publisher.

